# Mortality risk factors in actively resuscitated 22-week preterm infants: a case-control study focusing on NEC and maternal hospitalization

**DOI:** 10.1186/s12887-025-06178-3

**Published:** 2025-10-09

**Authors:** Tomonori Kurimoto, Takuya Tokuhisa, Keisuke Ohtsuka, Toshio Harumatsu, Hiroshi Ohashi, Eiji Hirakawa, Takatsugu Maeda, Masato Kamitomo

**Affiliations:** 1 Department of Neonatology, Perinatal Medical Center, Kagoshima City Hospital, 37-1 Uearata, Kagoshima, Japan; 2 Department of Obstetrics and Gynecology, Perinatal Medical Center, Kagoshima City Hospital, Kagoshima, Japan

**Keywords:** Extremely preterm infants, 22 weeks' gestation, Neonatal mortality, Necrotizing enterocolitis, Tension pneumothorax, Intraventricular hemorrhage, Bacteremia, Maternal hospitalization duration, Birth outside of tertiary centers

## Abstract

**Background:**

Infants born at 22 weeks’ gestation represent one of the most vulnerable populations in neonatal care. Despite increasing survival rates with active resuscitation, variability in outcomes remains significant. This study aimed to identify risk factors associated with in-hospital mortality in actively resuscitated infants born at 22 weeks’ gestation.

**Methods:**

A retrospective case-control study was conducted at Kagoshima City Hospital between January 2006 and December 2023. Fifty-seven live-born infants at 22 weeks’ gestation who received active resuscitation were included. Clinical characteristics and outcomes were compared between survivors and non-survivors. Univariable and multivariable logistic regression analyses were performed. Firth’s correction and cross-validation were used to address small sample size and model robustness.

**Results:**

Of the 57 infants included, 19 (33.3%) died during hospitalization. Necrotizing enterocolitis (NEC, stage ≥ II) and maternal hospitalization ≤ 5 days prior to delivery were independently associated with mortality (adjusted odds ratios: 5.4 and 6.4, respectively). Only non-survivors experienced tension pneumothorax or were born outside of tertiary centers. Bacteremia within 10 days and intraventricular hemorrhage grade III–IV were also associated with mortality in univariable analyses. Model performance after sensitivity analyses yielded an AUC of 0.892 and improved calibration.

**Conclusions:**

NEC and short maternal hospitalization are significant independent predictors of mortality in infants born at 22 weeks of gestation. Delivery at tertiary centers and timely maternal transfer may improve survival. Further studies are needed to establish preventive strategies for NEC, sepsis, and respiratory complications in this high-risk group.

**Supplementary Information:**

The online version contains supplementary material available at 10.1186/s12887-025-06178-3.

## Background

Infants born at 22 weeks’ gestation represent one of the highest-risk populations in neonatal care. Among live-born infants at this gestational age, reported survival to hospital discharge varies widely across countries, ranging from 3.7% to 56.7% [1]. In recent years, an increasing number of hospitals worldwide have adopted active resuscitation for these infants, contributing to gradual but notable improvements in survival rates [2, 3]. Despite these advances, substantial variability in outcomes persists, reflecting the absence of a standardized approach to management.

The care of infants born at 22 weeks involves complex clinical decision-making, and the factors contributing to mortality at this gestation are still unclear. This study aimed to identify specific risk factors associated with mortality in infants born at 22 weeks’ gestation, using a case-control analysis conducted at a tertiary care center. A better understanding of these factors may support the development of more effective interventions and improve survival outcomes for this extremely vulnerable population.

## Methods

### Study design and setting

This retrospective case–control study was conducted at Kagoshima City Hospital, a tertiary medical center that received maternal and neonatal transfers from primary and secondary perinatal care facilities in Kagoshima Prefecture.

### Study population and inclusion criteria

We included all infants born between 22 weeks and 0 days to 22 weeks and 6 days of gestation between January 1, 2006, and December 31, 2023, who were actively resuscitated and admitted to our NICU. Exclusion criteria included congenital anomalies, such as critical pulmonary stenosis (*n* = 1).

### Exposure definitions and variable classification

NEC was defined as Bell’s stage II or higher. Severe IVH was classified as grade III or IV. Bacteremia was defined by positive blood culture. SGA was defined as birth weight < 10th percentile based on Japanese charts. Maternal hospitalization duration was dichotomized using a cutoff of ≤ 5 days. Outborn status was defined as birth at a primary/secondary facility followed by postnatal transport.

All mothers at risk of delivering at 22 weeks’ gestation were transferred to our institution for specialized care. In cases where labor began before the mother could be transferred, neonatologists were dispatched to primary or secondary perinatal care facilities, where active resuscitation was initiated. The infants were then transported to our hospital for further management. Depending on maternal and fetal conditions, delivery was performed via cesarean section or vaginal delivery. The reasons for maternal hospitalization included threatened preterm labor due to cervical shortening and dilation, uterine contractions or vaginal bleeding as well as premature rupture of membranes. Active resuscitation was initiated regardless of birth weight or initial condition. Interventions included endotracheal intubation, ventilation optimization, and chest compressions. If the heart rate remained below 60 beats per minute despite these measures for 45–60 s, intravenous epinephrine (10–30 µg/kg per dose) was administered every 3 min. If intravenous access was unavailable, epinephrine (50–100 µg/kg per dose) was given via the endotracheal tube. Normal saline was administered when hypovolemia was suspected. All infants received surfactant therapy [4]. At our institution, the criteria for active resuscitation in 22-week infants remained consistent throughout the study period (2006–2023). Specifically, the placement of an endotracheal tube followed by administration of surfactant was considered the initiation of active resuscitation. All 22-week infants responded with return of spontaneous circulation following this intervention, and no substantial modifications were made to the institutional resuscitation protocols during the study period. Therefore, we did not perform stratified analysis by time period.

### Data collection

Data were collected from electronic medical records. Gestational age was estimated based on the last menstrual period, crown-rump length measurements, or a combination of both, as determined by early pregnancy ultrasound assessment. In cases where there was a discrepancy between the LMP and early ultrasound findings, the estimated due date was determined according to the ultrasound measurements. Antepartum stillbirth was defined as intrauterine fetal death prior to the onset of labor, while intrapartum stillbirth referred to fetal demise occurring during labor. Between 2006 and 2023, a total of 58 infants were delivered at 22 weeks of gestation. Nine stillbirths were reported. During this period, all live-born infants were actively resuscitated. One infant with congenital heart disease (critical pulmonary stenosis) was excluded. No other major congenital anomalies were identified. Maternal data, placental pathology, fetal heart rate (FHR) monitoring, and neonatal information were compared between infants who died during hospitalization and those who survived to discharge. No missing data were observed for variables included in the analysis.

### Definitions

A complete course of antenatal corticosteroids (ACS) was defined as the administration of at least two doses during the current pregnancy to promote fetal lung maturation. Abnormal FHR patterns were assessed according to the 2015 guidelines of the International Federation of Gynecology and Obstetrics. Bradycardia was defined as an FHR of used < 110 beats per minute (bpm) persisting for ≥ 10 min [5]. Recurrent late decelerations were defined as those occurring with ≥ 50% of uterine contractions [6]. Histopathological examination of the placenta and umbilical cord was performed to identify chorioamnionitis and funisitis, which were staged using the Blanc classification system [7]. Bacteremia was defined as the presence of microorganisms in blood cultures. Head and cardiac ultrasound examinations were performed by neonatologists within the first 24 h of life: three to four times on day 1, three times on days 2 and 3, and two to three times on day 4. Hemodynamic support with dopamine, dobutamine, or corticosteroids was administered based on clinical condition and echocardiographic findings. Patent ductus arteriosus was considered hemodynamically significant if confirmed by echocardiography and treated pharmacologically [8].

Intraventricular hemorrhage (IVH) was graded using the Volpe classification [9]. Meconium-related ileus was diagnosed intraoperatively when a microcolon or narrow colon extended to the distal ileum, accompanied by dilatation of the proximal ileum filled with thick meconium and a gradual caliber change [10]. Focal intestinal perforation was diagnosed based on surgical findings of a punched-out perforation without surrounding necrosis [11]. Necrotizing enterocolitis (NEC) was defined as Bell’s stage II or higher, according to the Modified Bell’s Staging Criteria [12]. For infants with Bell’s stage III NEC who underwent surgery, the diagnosis was histopathologically confirmed following surgical intervention. Tension pneumothorax was diagnosed by radiographic findings including lung displacement from the chest wall, diaphragmatic depression, contralateral mediastinal shift, or radiolucency along the posterior axillary line, and required thoracentesis and chest tube insertion. Infants with birth weights below the 10th percentile were classified as small for gestational age (SGA) based on Japanese reference charts. All infants received antibiotics and antifungal agents at birth. Oral miconazole was administered beginning in the third postnatal week to prevent NEC [13]. Postnatal management—including resuscitation, insertion of umbilical arterial and venous catheters, fluid and electrolyte management, nutrition, and environmental control (humidity and temperature) in the incubator—was performed according to our hospital’s previously published protocol [14].

### Outcomes

The primary outcome was the risk of mortality with infants born at 22 weeks.

### Statistical analyses

Differences between the non-survivor and survivor groups were assessed using the chi-square test or Fisher’s exact test for categorical variables. Continuous variables were non-normally distributed and therefore analyzed using the Mann–Whitney U test. As this was a retrospective study including all eligible cases during the study period, no a priori sample size calculation or power analysis was performed. Two-sided p-value of < 0.05 were considered statistically significant. Where applicable, 95% confidence intervals (CIs) were reported. To evaluate associations between clinical variables and in-hospital mortality, univariable logistic regression analyses were conducted.

A receiver operating characteristic (ROC) curve was constructed to assessed the predictive performance of maternal hospitalization duration. Based on this analysis, a cutoff value of ≤ 5 days was identified and applied in subsequent analyses.

In-hospital mortality was defined as the outcome variable. Given the limited number of events (*n* = 19), the number of explanatory variables included in the multivariable logistic regression model was restricted to two to reduce the risk of overfitting. NEC (stage ≥ II) and maternal hospitalization duration were selected based on their statistical significance in univariable analyses and clinical relevance to early intervention and prognosis.

Given the small number of outcome events (*n* = 19), Firth’s penalized logistic regression was selected to mitigate small-sample bias and provide stable estimates. A two-variable model was chosen to avoid overfitting and ensure interpretability. Although alternative methods such as elastic-net regression or Bayesian shrinkage could have accommodated more variables (e.g., IVH or early-onset sepsis), these approaches require either larger sample sizes or strong prior assumptions, which were not feasible in our dataset.

Although severe IVH and bacteremia within 10 days were also significant in univariable models, they were excluded from the multivariable analysis. IVH was excluded due to the potential for reverse causality (i.e., severe clinical deterioration may lead to IVH). Bacteremia occurred within the first 10 days of life, whereas NEC (stage ≥ II) occurred only after day 11 (median day of onset: day 63; interquartile range [IQR]: days 49–81). Despite its statistical significance and temporal independence from NEC (stage ≥ II), bacteremia within 10 days of birth was excluded due to its low frequency (four cases in the non-survivor group and one in the survivor group), which posed a risk of overfitting. Tension pneumothorax and birth at non-tertiary perinatal care facilities were also excluded from the multivariable analysis due to complete separation, which would have led to unstable or infinite OR estimates. To explore potential confounding factors, additional models adjusting for SGA status and one course of antenatal steroid administration were constructed. To assess multicollinearity, variance inflation factors (VIFs) were calculated. The predictors maternal hospitalization ≤ 5 days and NEC (stage ≥ Ⅱ)VID each had a VIF of 1.02, suggesting negligible multicollinearity. Additionally, the VIF for outborn status was below 2.0, further supporting the absence of concerning collinearity among predictors.　The VIF for the constant term was 3.60, which is within acceptable limits. However, the events per variable (EPV) in the multivariable model was less than 5, which may lead to wide confidence intervals and potential instability in the regression estimates. This limitation should be taken into account when interpreting the results. All logistic regression models were estimated using the maximum likelihood method. Statistical analyses were performed using R (R Foundation for Statistical Computing, Vienna, Austria) and SAS version 14.1 (SAS Institute Inc., Cary, NC, USA). Model performance was assessed using stratified 5-fold cross-validation. Discriminative ability was evaluated using the area under the ROC curve (AUC) with 95% CI, sensitivity, and specificity. Calibration was assessed using calibration plots and the Brier score. Model robustness was evaluated via sensitivity analyses by identifying and excluding influential observations using Cook’s distance (Cook’s D); observations with Cook’s D > 4/n were considered influential. Model performance metrics, including AUC, sensitivity, specificity, and calibration were re-assessed post-exclusion using stratified 5-fold cross-validation, including AUC, sensitivity, specificity, and calibration metrics.

## Results

### In-hospital morbidity and mortality

Thirty-eight infants (66.7%) with 22 weeks of birth survived until discharge, and 19 infants (33.3%) passed away in the hospital. Tension pneumothorax occurred in five patients (26.3%) in the non-survivor group and none in the survivor group. NEC (stage ≥ II) occurred in seven patients (36.8%) in the non-survivor group and one patient (2.6%) in the survivor group. Severe IVH occurred in nine patients (47.4%) in the non-survivor group and seven patients (18.4%) in the survivor group. Bacteremia within 10 d after birth occurred in four patients (21.1%) in the non-survivor group and one patient (2.6%) in the survivor group.

**Fig. 1:**
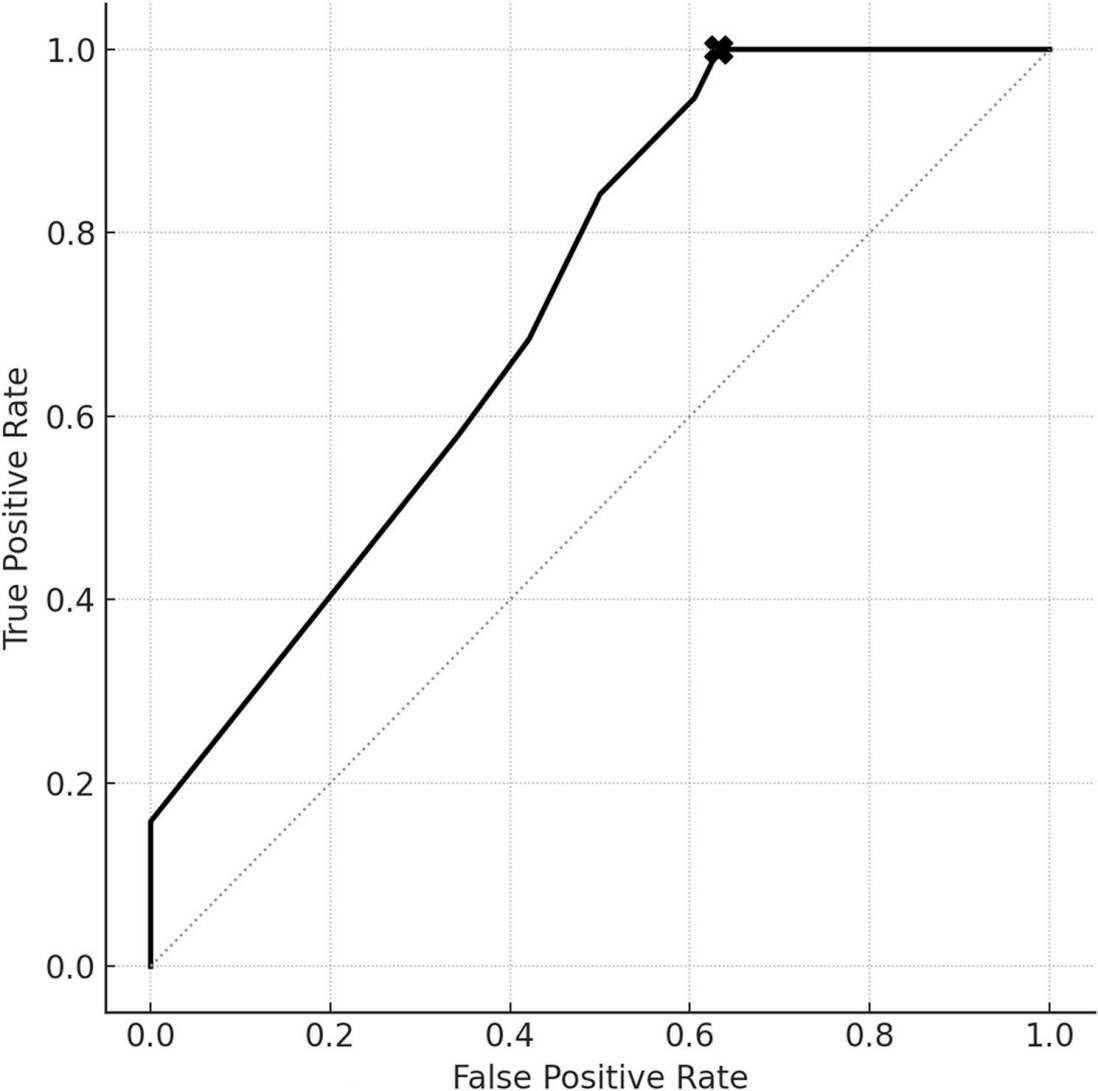
ROC Curve for predicting mortality based on reversed hospitalization duration. The ROC curve illustrates the predictive ability of maternal hospitalization duration (reversed) for neonatal mortality. The optimal cutoff value (≤ 5 days) was selected based on the Youden Index (sensitivity + specificity − 1). This threshold provided a sensitivity of 1.0 and specificity of 0.37, yielding a Youden Index of 0.37, and reflects a high true positive rate with limited specificity. The AUC was 0.724 (95% CI, 0.60–0.84), indicating moderate discriminative ability. ROC analysis was performed using standard nonparametric estimation with 95% confidence intervals. These results highlight the clinical relevance of short maternal stay as a potential early warning sign for neonatal risk. Abbreviations: AUC, area under the curve; CI, confidence interval; ROC, receiver operating characteristic; Youden Index, sensitivity + specificity − 1

The length of maternal hospitalization before delivery in the non-survivor group was 2 d (IQR: 1–3 d), and that in the survivor group was 5 d (IQR: 1–9 d) (Table 1). A receiver operating characteristic (ROC) curve was constructed to evaluate the association between the duration of maternal hospitalization before delivery and neonatal mortality. The analysis identified a threshold of ≤ 5 days of maternal hospitalization associated with increased neonatal mortality. The sensitivity and specificity of this threshold were 1.0 and 0.37, respectively. The corresponding false positive rate was 0.63. The Youden Index was 0.37. The area under the ROC curve (AUC) was 0.724 (95% CI, 0.60–0.84) (Fig. 1).

**Table 1:** Comparison of perinatal and clinical characteristics between non-survivor and survivor groups

Variable	Non-survivor group (*n* = 19)	Survivor group (*n* = 38)	*p*-value
Sex (male)	9 (47.4)	19 (50.0)	1
Birth weight (median)	484 [430–527]	493 [452–552]	0.43
FGR (10%tile)	2 (10.5)	4 (10.5)	1
Twin	4 (21.1)	10 (26.3)	0.75
Birth at non-tertiary hospital	3 (15.8)	0	0.03
Maternal age	32 [27–35]	32 [28–34]	0.61
Primipara	13 (68.4)	27 (71.1)	1
Cesarean delivery	8 (42.1)	23 (60.5)	0.26
P/D	0	2 (5.3)	0.55
V/D	3 (15.8)	7 (18.4)	1
Recurrent L/D	0	2 (5.3)	0.55
Bradycardia (FHR)	0	1 (2.6)	1
Tachycardia (FHR)	0	3 (7.9)	0.54
MgSO_4_	9 (47.4)	23 (60.5)	0.40
Ritodrine hydrochloride	11 (57.9)	28 (73.7)	0.24
Antibiotics	2 (10.5)	13 (34.2)	0.06
One course of ACS	2 (10.5)	9 (23.7)	0.30
ACS (within 24 h before birth)	3 (15.8)	5 (13.2)	1.0
ACS (within 7days before birth)	5 (26.3)	14 (36.8)	0.56
Threatened preterm labor	15 (78.9)	32 (84.2)	0.62
PROM	6 (31.6)	8 (21.1)	0.52
CAM stage 2–3	10 (52.6)	19 (50.0)	1
Funisitis stage 2–3	2 (10.5)	6(15.8)	0.71
HDP	0	1 (2.6)	1
Abruption	0	5 (13.2)	0.16
Fertility treatment	1 (5.3)	5 (13.2)	0.65
Maternal hospitalization duration	2 [1–3]	5 [1–9]	0.01
APS 1 min	2 [1–3]	2 [1–4]	0.68
APS 5 min	6 [4–6]	6 [5–7]	0.11
UApH	7.30 [7.24–7.37]	7.32 [7.27–7.38]	0.47
Tension pneumothorax	5 (26.3)	0	0.01
MRI	0	2 (5.3)	0.55
SIP	4 (21.1)	4 (10.5)	0.42
NEC (stage ≥ Ⅱ)	7 (36.8)	1 (2.6)	0.001
IND/IBU	14 (73.7)	32 (84.2)	0.48
IVH grade Ⅲ-Ⅳ	9 (47.4)	7 (18.4)	0.03
Bacteremia ≦ 10day	4 (21.1)	1 (2.6)	0.04
Antibiotics treatment duration	6 [4–8]	6 [6–8]	0.79
Day of first enteral feeding	4 [3–5]	4 [3–6]	0.20
Type of first feeding: breast milk	3(15.8)	4(10.5)	0.68

Values are presented as median [IQR] or *n* (%). Statistical significance was set at *p* < 0.05

Three neonates born at primary or secondary perinatal facilities died despite aggressive resuscitation and transfer to our hospital. Detailed case descriptions are provided in Supplementary Material (Supplementary Case Descriptions). Among the 19 non-survivors, 12 died without NEC due to other causes such as tension pneumothorax (*n* = 5), bacteremia (*n* = 3), spontaneous intestinal perforation (*n* = 3), and combined bacteremia and SIP (*n* = 1). Among the 19 non-survivors, 12 died without developing NEC. In these cases, other major complications observed around the time of death included tension pneumothorax (*n* = 5), bacteremia (*n* = 3), spontaneous intestinal perforation (*n* = 3), and combined bacteremia and SIP (*n* = 1). Among these 12 infants, six also exhibited severe intraventricular hemorrhage (grade III–IV).

### Multivariate analysis

In the multivariable logistic regression analysis (Table 2), NEC (stage ≥ Ⅱ), bacteremia within 10 days after birth, and IVH (grade 3-4) were independently associated with an increased risk of in-hospital mortality. In contrast, a longer duration of maternal hospitalization was significantly associated with a decreased risk of mortality.

**Table 2:** Univariable logistic regression analyses of factors associated with in-hospital mortality

Variable	OR [95%CI]	*p*-value
NEC (stage ≥ Ⅱ)	13.2 [1.4-123.3]	0.02
Maternal hospitalization	0.67 [0.49–0.92]	0.01
Bacteremia ≤ 10 days after birth	9.9 [1.02–95.7]	0.048
IVH grade 3–4	4.0 [1.2–13.5]	0.03

*Abbreviations*: *CI* confidence interval, *NEC* necrotizing enterocolitis, *OR* odds ratio, *IVH* intraventricular hemorrhage

ORs, 95% CIs, and p-values were calculated using univariable logistic regression. Variables significantly associated with in-hospital mortality included NEC (stage ≥ II), maternal hospitalization duration, bacteremia within 10 days after birth, and severe IVH (grade III–IV)

Variables such as tension pneumothorax and birth at non-tertiary perinatal care facilities were excluded from the analysis due to complete separation (i.e., all affected cases resulted in death), which would yield infinite or unstable ORs

The adjusted odds ratio (aOR) and 95% CI for NEC (stage ≥ II) were aOR 5.4 (1.1–25.5), and for maternal hospitalization duration of ≤ 5 d were aOR 6.4 (1.6–25.1) in a Firth logistic regression model (Table 3).

**Table 3:** Firth logistic regression analyses for factors associated with in-hospital mortality

Variable	OR [95%CI]	*p*-value	aOR [95%CI]	*p*-value
NEC (stage ≥ Ⅱ)	5.3 [1.1–25.1]	0.04	5.4 [1.1–25.5]	0.04
Maternal hospitalization ≤ 5days	6.4 [1.6–24.8]	0.01	6.4 [1.6–25.1]	0.01

*Abbreviations*: *OR* odds ratio, *aOR* adjusted odds ratio, *CI* confidence interval, N*EC* necrotizing enterocolitis, *SGA* small for gestational age

ORs and aORs with 95% CIs and *p*-values are presented. Variables included in the multivariable model—NEC (stage ≥ II) and maternal hospitalization duration ≤ 5 days—were selected based on statistical significance in univariable analyses and clinical relevance. To explore potential confounding, additional models adjusting for SGA status and receipt of a single course of antenatal steroid administration were constructed. Both NEC and short maternal hospitalization remained significantly associated with in-hospital mortality in the adjusted model

### Model performance

The Firth logistic regression model was evaluated using stratified 5-fold cross-validation. The mean accuracy was 0.755 ± 0.062. The area under the ROC AUC was 0.746 (95% CI: 0.601–0.868), calculated using 1,000 bootstrap resamples. The optimal cutoff value determined by the Youden Index was 0.338, corresponding to a sensitivity of 0.316 and specificity of 0.974 (Table 4).

**Table 4:** Comparison of model performance before and after exclusion of influential observations identified by cook’s distance

Variable	Before	After
Accuracy (± SD)	0.755 ± 0.062	0.824 ± 0.039
Sensitivity	0.316	0.36
Specificity	0.974	1
AUC [95%CI]/ROC	0.746 [0.601–0.868]	0.892 [0.791–0.965]
cut off value	0.338	0.397
Brier score	0.177	

*Abbreviations*: *ROC AUC* receiver operating characteristic area under the curve, *Cook’s D* Cook’s distance, *CI* confidence interval *SD* standard deviation

Model performance metrics before and after exclusion of six influential observations (Cook’s D > 4/*n* = 0.070). Accuracy is presented as mean ± SD from stratified 5-fold cross-validation. Discrimination was evaluated using sensitivity, specificity, and the area under the ROC AUC with 95% CI. Calibration was assessed using the Brier score. The optimal cutoff probability for classification was determined using the Youden index. After exclusion, improvements were observed in all metrics, particularly AUC and specificity

The calibration plot demonstrated good agreement between the predicted probabilities and the observed incidence of death. Across risk deciles, the predicted probabilities were generally close to the actual outcomes, with no notable overestimation or underestimation. The Brier score for the model was 0.177, indicating acceptable overall model calibration.

**Fig. 2:**
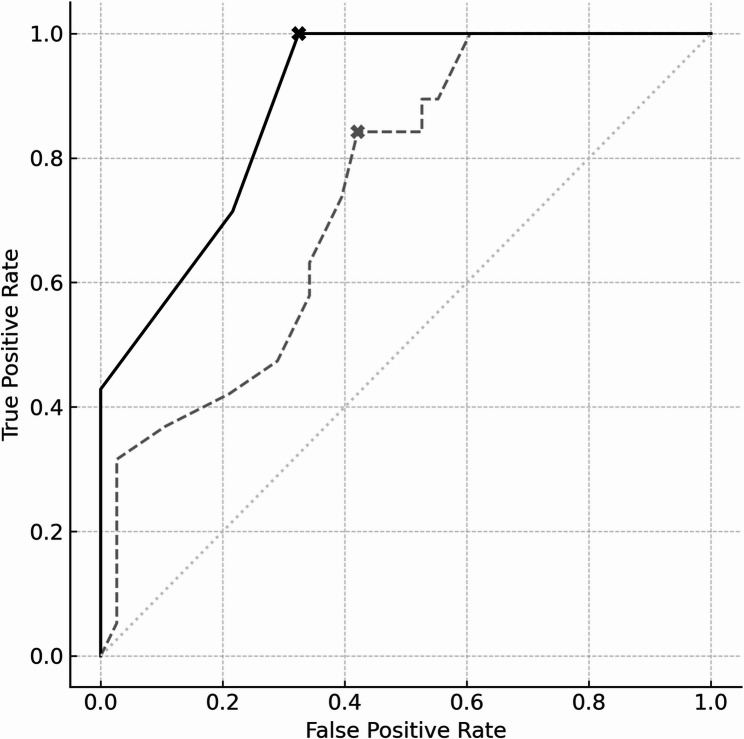
Comparison of firth logistic regression model performance before and after exclusion of influential observations. Receiver operating characteristic (ROC) curves compare the performance of the Firth logistic regression model before (gray dashed line) and after (black solid line) exclusion of influential observations, identified by Cook’s distance (Cook’s D > 0.070). Cutoff values for classification were determined based on the Youden Index, which shifted from 0.338 in the original model to 0.397 after exclusion. The original model had an AUC of 0.746 (95% CI: 0.601–0.868), sensitivity of 0.316, and specificity of 0.974. After removal of six influential cases, the AUC improved to 0.892 (95% CI: 0.791–0.965), specificity reached 1.0, and sensitivity increased to 0.36. ROC analysis was based on nonparametric estimation methods. Abbreviations: AUC, area under the curve; CI, confidence interval; Cook’s D, Cook’s distance; ROC, receiver operating characteristic; Youden Index, sensitivity + specificity − 1

**Fig. 3:**
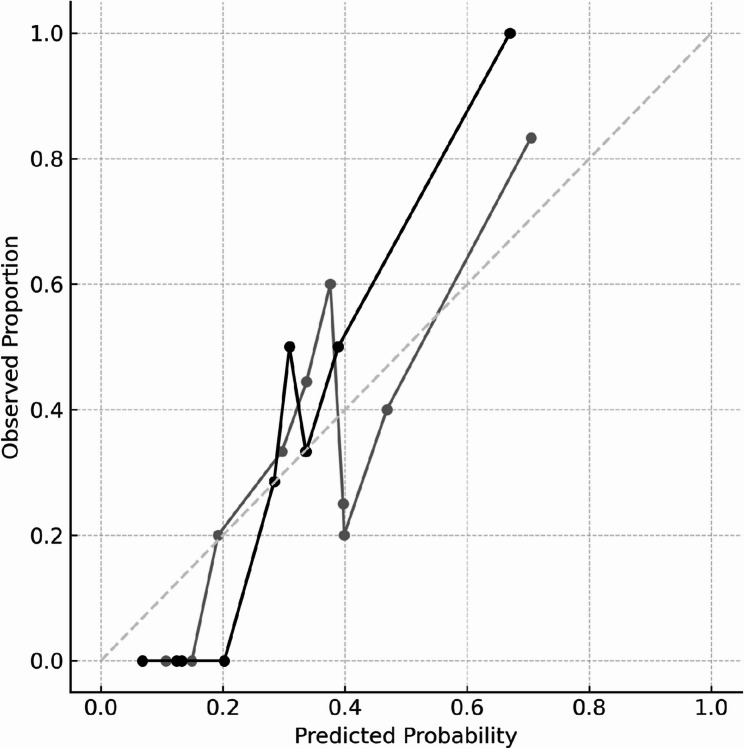
Calibration of the firth logistic regression model before and after exclusion of influential observations. Calibration plots showing the relationship between predicted probabilities and observed proportions for the Firth logistic regression model before (gray line) and after (black line) exclusion of influential observations, identified using Cook’s distance (> 4/n). The diagonal dashed line represents perfect calibration. After exclusion, the model demonstrated improved calibration, with the black line aligning more closely with the diagonal, indicating better agreement between predicted and observed mortality risks. Abbreviations: Cook’s D, Cook’s distance

To assess model robustness, Cook’s D was calculated. Six influential observations were identified, with Cook’s D values exceeding the threshold of 4/n (0.070); the maximum Cook’s D was 0.451. These observations were excluded in a secondary analysis. After exclusion of the influential cases, the model’s performance improved. The AUC increased to 0.892 (95% CI: 0.791–0.965), the optimal cutoff value increased to 0.338 and accuracy rose from 0.755 to 0.824. Specificity improved from 0.974 to 1.0, while sensitivity showed a modest increase from 0.316 to 0.357 (Fig. 2). Calibration plots also indicated enhanced alignment between predicted and observed probabilities following the exclusion, suggesting better model fit (Fig. 3).

## Discussion

This study evaluated short-term outcomes and mortality-associated factors in actively resuscitated infants born at 22 weeks of gestation. Among 57 included infants, 66.7% survived to discharge, while 33.3% died during hospitalization. Multivariable analysis identified NEC (stage ≥ II) and a maternal hospitalization duration of ≤ 5 days prior to delivery as independent predictors of in-hospital mortality. Additionally, tension pneumothorax and birth at non-tertiary perinatal care facilities were associated with universally fatal outcomes, though excluded from multivariable modeling due to complete separation.

### NEC

In our study, NEC (stage ≥ II) was significantly associated with in-hospital mortality among infants born at 22 weeks of gestation. Despite the prophylactic use of miconazole since 2002 at our institution, the incidence of NEC remained notably high in this population [13]. This underscores the persistent vulnerability of the immature intestinal tract at this gestational age.　The role of probiotics in NEC prevention remains controversial. A recent systematic review and meta-analysis concluded that probiotics do not significantly reduce either mortality or the incidence of severe NEC in extremely preterm infants [15, 16]. At our institution, probiotics are not routinely administered, owing to the lack of consensus regarding optimal strain selection, dosing strategies, and long-term safety.

In contrast, the benefits of donor human milk are well established. A meta-analysis demonstrated a significantly lower risk of NEC among infants receiving donor human milk compared to those fed with formula (risk ratio: 0.53, 95% CI: 0.35–0.81) [17]. However, donor human milk was not available at our institution during the study period, and therefore was not used in any case. Moreover, the rate of exclusive maternal breast milk as the first enteral feeding was low in both the non-survivor (15.8%) and survivor (10.5%) groups (*p* = 0.68), reflecting the practical limitations of early enteral nutrition in this population. While human milk has been shown to promote intestinal maturation, regulate the gut microbiota, and enhance immune defense mechanisms, the absence of donor milk access may have contributed to the high NEC rate in our cohort [18]. The incorporation of donor human milk into feeding protocols may be a promising strategy for NEC prevention in the future, particularly for institutions caring for extremely preterm infants.

### Maternal hospitalization of duration

Our study found that shorter maternal hospitalization (≤ 5 days) was independently associated with NEC-related mortality. Limited inpatient time may preclude adequate antenatal interventions, including timely administration of ACS, infection screening, and management of premature rupture of membranes or intrauterine infection [19]. These perinatal factors can impair fetal intestinal perfusion and immune readiness, predisposing neonates to NEC after birth.

### Tension pneumothorax

Tension pneumothorax was observed exclusively in the non-survivor group and was universally fatal, highlighting its critical role as a predictor of early neonatal death. This finding is consistent with our previous report, which demonstrated that tension pneumothorax frequently occurs within 72 h of birth in neonates born at 22–23 weeks of gestation [20].　High-frequency oscillatory ventilation (HFOV) is routinely initiated soon after birth in some institutions, particularly for extremely preterm infants, based on the rationale that it may reduce ventilator-induced lung injury [21, 22]. However, HFOV has also been associated with an increased risk of pulmonary air leaks, including tension pneumothorax, in this vulnerable population [23].　Although volume-guarantee (VG) ventilation has been proposed as a lung-protective strategy, its efficacy in preventing air leak syndromes, particularly in neonates born at 22–23 weeks, remains inconclusive due to limited data [24]. In our study, synchronized intermittent mandatory ventilation (SIMV) was employed following surfactant administration, in accordance with current Japanese perinatal practice guidelines [25].　Given the extreme fragility of the immature alveoli in this gestational age group, post-surfactant ventilatory management must be individualized and meticulously adjusted to minimize the risk of barotrauma and subsequent complications such as pneumothorax. Early detection and intervention remain essential in improving outcomes.

### Bacteremia within 10 d after birth

In this study, bacteremia occurring within 10 days after birth was significantly associated with in-hospital mortality in univariable analysis. This finding is consistent with previous reports indicating that early and late-onset sepsis increases the risk of mortality and adverse neurodevelopmental outcomes in extremely preterm infants [26, 27].

Although prophylactic antibiotics and antifungals are commonly used in Japanese NICUs, prolonged antibiotic exposure beyond 5 days has been linked to an increased risk of NEC [25, 28]. Standardized protocols for timely discontinuation based on negative culture results may help reduce such risks.

Central line-associated bloodstream infections remain a concern despite adherence to aseptic techniques during catheter insertion [29]. While some catheter modifications, such as silver-impregnated lines, have shown promise in reducing infection risk [30], the evidence is insufficient. Further studies are warranted to determine optimal catheter strategies and antibiotic durations in this vulnerable population [31–33].

### Antenatal corticosteroids

Although ACS have been shown to improve neonatal survival at 22 weeks of gestation, the rate of complete ACS administration in our study was low [34]. This likely reflects institutional differences in perinatal care approaches, challenges with the timing of labor, and limitations in decision-making at the threshold of viability [35–37]. In many cases, rapid progression of preterm labor or uncertainty surrounding the prognosis at 22 weeks may hinder timely administration [34]. To improve neonatal outcomes, early maternal admission, standardized perinatal protocols, and proactive decision-making are essential to ensure adequate ACS exposure in this high-risk group [37].

### Birth at non-tertiary perinatal care facilities

All three infants born at non-tertiary centers died despite intensive resuscitation and transport, each presenting with severe IVH on admission. These results align with prior studies indicating that antenatal maternal transfer, rather than postnatal neonatal transport, is associated with reduced mortality and IVH incidence in extremely preterm infants [38, 39]. In contrast, neonatal transport introduces physiological instability due to prolonged handling, suboptimal ventilation during transfer, and delayed access to advanced care, thereby increasing the risk of complications such as hypoxia and IVH [40].

These findings align with a previous report suggesting that all infants born at 22 weeks of gestation who underwent neonatal transport experienced fatal outcomes [41]. Collectively, these cases reinforce that antenatal maternal transfer remains critical for infants at 22 weeks of gestation, ensuring immediate access to tertiary-level care and potentially improving survival and neurodevelopmental outcomes.

### Clinical implications and modifiable factors

NEC (stage ≥ Ⅱ) and short maternal hospitalization (≤ 5 days) were independent predictors of in-hospital mortality. While NEC reflects intrinsic vulnerability, shorter hospitalization likely limited opportunities for antenatal interventions such as corticosteroid administration or infection screening.

In addition, all neonates born outside tertiary centers died despite intensive resuscitation, emphasizing the importance of antenatal maternal transfer over neonatal transport. These findings highlight modifiable system-level factors—such as early maternal admission and standardized referral protocols—as key targets to improve outcomes at 22 weeks of gestation.

### Limitations

This study has several limitations. First, it was conducted at a single tertiary center, which may limit the generalizability of the findings to other settings with different perinatal care practices. However, to improve internal validity, we applied Firth’s penalized logistic regression and stratified 5-fold cross-validation to address small sample size and reduce model overfitting.

Second, the study spanned 18 years (2006–2023), during which clinical practices such as antenatal corticosteroid administration, delivery room resuscitation, and nutritional protocols may have evolved. These changes in perinatal management could have influenced both mortality and morbidity outcomes. While we standardized care as much as possible based on institutional protocols, temporal variations remain a potential source of bias.

Third, due to its retrospective nature, the study may be subject to information bias and unmeasured confounding. While sensitivity analyses excluding influential observations (based on Cook’s distance) were performed and showed improved model performance, residual bias cannot be excluded.

Fourth, the number of in-hospital deaths was 19, resulting in fewer than five EPV in the regression models. This may lead to wide confidence intervals and potential model instability, which should be considered when interpreting the findings.

Fifth, although alternative variable selection methods such as elastic-net regression or Bayesian shrinkage approaches could have been considered, we chose a two-variable Firth logistic regression model due to concerns about overfitting, model convergence, and our intent to include variables such as IVH and early-onset bacteremia that may introduce bias due to reverse causality or low frequency.

Sixth, although stratified 5-fold cross-validation was used to evaluate model performance, the small sample size (*n* = 57) limits the stability and generalizability of the AUC estimates. Each fold included approximately 11–12 cases, which may result in variability in performance metrics. The bootstrap-0.632 method could be considered in future studies to obtain more robust estimates of predictive accuracy.

Seventh, our study included multiple sets of twins, but clustering due to non-independence between twins was not statistically adjusted. This may have led to underestimation of standard errors, and the assumption of independence should be interpreted with caution.

Eighth, the small cohort size limited the statistical power to detect associations with less frequent variables. Larger multicenter prospective studies are needed to validate these findings and further explore strategies to improve outcomes for infants born at 22 weeks of gestation.

Ninth, all infants in this study received active resuscitation based on parental wishes, as part of a shared decision-making process. While this ensured consistent care within our cohort, it may limit comparability to institutions where selective resuscitation is practiced based on institutional policy or estimated prognosis. Parental preferences and institutional culture may thus act as confounding factors when interpreting international or inter-facility differences.

Tenth, we used a 5-day threshold for antenatal maternal hospitalization to dichotomize the variable. This cutoff was chosen a priori based on clinical rationale and logistical considerations in Japanese perinatal practice. However, the threshold was not optimized through cross-validation or external validation, and therefore may introduce cutoff-related bias. This limitation should be considered when interpreting the predictive value of this variable, and future validation studies are warranted.

## Conclusion

NEC and maternal hospitalization ≤ 5 days were independently associated with in-hospital mortality in infants born at 22 weeks of gestation. Early bacteremia and severe IVH were also linked to mortality in univariable analysis. Tension pneumothorax and birth outside of tertiary centers occurred only in non-survivors, precluding regression analysis but highlighting their clinical relevance.

These findings underscore the importance of early maternal transfer and delivery at tertiary care centers. Further research is needed to develop strategies to prevent NEC, sepsis, and ventilation-related complications in this extremely high-risk population.

## Supplementary Information

Supplementary Material 1.

Supplementary Material 2.

## Data Availability

The datasets generated and/or analyzed during the current study are available from the corresponding author on reasonable request.

## Abbreviations

ACS: Antenatal corticosteroids
AUC: Area under the curve
CI: Confidence interval
FHR: Fetal heart rate
HFOV: High-frequency oscillatory ventilation
IQR: Interquartile range
IVH: Intraventricular hemorrhage
NEC: Necrotizing enterocolitis
NICU: Neonatal intensive care unit
OR: Odds ratio
ROC: Receiver operating characteristic
RCT: Randomized controlled trial
SD: Standard deviation
SGA: Small for gestational age
SIMV: Synchronized intermittent mandatory ventilation
VG: Volume-guarantee ventilation
VIF: Variance inflation factor
