# Supplementary material for: Mortality risk factors in actively resuscitated 22-week preterm infants: a case-control study focusing on NEC and maternal hospitalization
Source: BMC Pediatr. 2025 Oct 9;25:788. doi: 10.1186/s12887-025-06178-3 (PMC12513028; doi:10.1186/s12887-025-06178-3)
Supplement: Supplementary file 1 — Supplementary Material 1. [file 12887_2025_6178_MOESM1_ESM.docx]

**Detailed Case Descriptions of Neonates Born at Non-tertiary Facilities**

**Case 1: Birth at Secondary Perinatal Facility (22 weeks and 5 days)**

The mother presented to a primary perinatal facility with preterm premature rupture of membranes at home. The fetal foot was protruding through the external uterine orifice. Due to the geographic distance from a tertiary center, she was transferred to a nearby secondary perinatal facility. Our hospital’s neonatal and obstetric team was dispatched by helicopter. Upon arrival, the fetal buttocks were present in the vaginal canal, and vaginal breech delivery ensued. The neonate received intubation, surfactant administration, and umbilical catheterization, and was transported to our hospital. On arrival, the neonate was diagnosed with grade III intraventricular hemorrhage (IVH).

**Case 2: Birth at Primary Perinatal Facility (21 weeks and 6 days)**

The mother experienced preterm premature rupture of membranes and was brought to a primary perinatal facility. The fetus showed repeated prolonged decelerations (nadir, 60 bpm; duration, 3–4 minutes), and the cervix was fully dilated. Transfer was not feasible. A neonatologist from our hospital was dispatched. Vaginal delivery was performed. Immediate resuscitation with intubation and surfactant administration was provided. After establishing umbilical access, the neonate was transferred to our NICU. Initial imaging revealed grade IV IVH.

**Case 3: Birth at Secondary Perinatal Facility (22 weeks and 5 days)**

The mother presented in labor to a primary perinatal facility. The internal uterine orifice was fully dilated with bulging membranes. Due to the risk of delivery during transport, she was transferred to a secondary facility. A neonatologist was dispatched for on-site support. The fetus was delivered vaginally with bradycardia (HR ~20 bpm). Resuscitation and stabilization were initiated, including surfactant administration and umbilical access. During transport, the neonate experienced seizures. Upon NICU admission, grade III IVH was diagnosed. Glucose solution, catecholamines, and sedatives were administered during transport.

**Notes**

All three neonates died despite receiving aggressive resuscitation. In each case, neonatologists were present at delivery. However, due to poor postnatal adaptation, endotracheal intubation, surfactant administration, vascular access placement, and administration of 10% glucose and catecholamines were required at the referring facility. The infants were stabilized in incubators and transferred to our NICU, arriving approximately 40–50 minutes after birth. Upon arrival, all three neonates exhibited physiological instability, highlighting the vulnerability associated with pre-delivery critical conditions, emergency resuscitation in non-tertiary settings, postnatal transport even under well-coordinated conditions, and the extreme prematurity of the infants at birth.

**Table 1. Comparison of clinical characteristics between PROM and non-PROM groups**

| **Variable** | **PROM group**  **(n=14)** | **Non-PROM group**  **(n=43)** | **p-value** |
| --- | --- | --- | --- |
| **Non-survivor** | 6 (42.9) | 13 (30.2) | 0.52 |
| **IVH grade Ⅲ-Ⅳ** | 3 (21.4) | 13 (30.2) | 0.73 |
| **Tension pneumothorax** | 2 (14.3) | 3 (7.0) | 0.59 |
| **NEC (stage ≥ Ⅱ)** | 3(21.4) | 5 (11.6) | 0.39 |
| **Bacteremia ≦ 10day** | 2 (14.3) | 3 (7.0) | 0.59 |

**Abbreviations:** IVH, intraventricular hemorrhage; NEC, necrotizing enterocolitis.

**Table 2. Comparison of clinical characteristics between CAM stage 2-3 and non-CAM stage 2-3 groups**

| **Variable** | **CAM stage 2-3 group (n=29)** | **Non-CAM stage 2-3 group (n=28)** | **p-value** |
| --- | --- | --- | --- |
| **Non-survivor** | 10 (34.5) | 9 (32.1) | 1.0 |
| **IVH grade Ⅲ-Ⅳ** | 6 (20.7) | 10 (35.7) | 0.25 |
| **Tension pneumothorax** | 3 (10.3) | 2 (7.1) | 1.0 |
| **NEC (stage ≥ Ⅱ)** | 4 (13.8) | 4 (14.3) | 1.0 |
| **Bacteremia ≦ 10day** | 2 (6.9) | 3 (10.7) | 0.67 |

**Abbreviations:** IVH, intraventricular hemorrhage; NEC, necrotizing enterocolitis.

**Table 3. Comparison of clinical characteristics between cesarean section and vaginal delivery groups**

| **Variable** | **Cesarean section group (n=31)** | **Vaginal delivery group**  **(n=26)** | **p-value** |
| --- | --- | --- | --- |
| **Non-survivor** | 8 (25.8) | 11 (42.3) | 0.26 |
| **IVH grade Ⅲ-Ⅳ** | 11 (35.5) | 5 (19.2) | 0.24 |
| **Tension pneumothorax** | 3 (9.7) | 2 (7.7) | 1.0 |
| **NEC (stage ≥ Ⅱ)** | 3 (9.7) | 5 (19.2) | 0.45 |
| **Bacteremia ≦ 10day** | 2 (6.5) | 3 (11.5) | 0.65 |

**Abbreviations:** IVH, intraventricular hemorrhage; NEC, necrotizing enterocolitis.

**Table 4. Comparison of clinical characteristics between threatened preterm labor and non- threatened preterm labor groups**

| **Variable** | **Threatened preterm labor group (n=47)** | **Non- Threatened preterm labor group (n=10)** | **p-value** |
| --- | --- | --- | --- |
| **Non-survivor** | 15 (31.9) | 4 (40.0) | 0.72 |
| **IVH grade Ⅲ-Ⅳ** | 14 (29.8) | 2 (20.0) | 0.71 |
| **Tension pneumothorax** | 3 (6.4) | 2 (20.0) | 0.21 |
| **NEC (stage ≥ Ⅱ)** | 6 (12.8) | 2 (20.0) | 0.62 |
| **Bacteremia ≦ 10day** | 4 (8.5) | 1 (10.0) | 1.0 |

**Abbreviations:** IVH, intraventricular hemorrhage; NEC, necrotizing enterocolitis.

**Table 5. Comparison of clinical characteristics between ACS administration (within 7 days) and non- ACS administration groups.**

|  | **ACS group (n=19)** | **Non- ACS group (n=38)** | **p-value** |
| --- | --- | --- | --- |
| **Non-survivor** | 5 (26.3) | 14 (36.8) | 0.43 |
| **IVH grade Ⅲ-Ⅳ** | 5 (26.3) | 11 (28.9) | 0.84 |
| **Tension pneumothorax** | 1 (5.3) | 4 (10.5) | 0.51 |
| **NEC (stage ≥ Ⅱ)** | 3 (15.8) | 5 (13.2) | 0.79 |
| **Bacteremia ≦ 10day** | 1 (5.2) | 4 (10.5) | 0.51 |

**Abbreviations:** ACS, antenatal corticosteroids; IVH, intraventricular hemorrhage; NEC, necrotizing enterocolitis.

**Table 6. Firth Logistic Regression analyses for factors associated with in-hospital mortality.**

| **Variable** | **OR [95%CI]** | **p-value** | **aOR [95%CI]** | **p-value** |
| --- | --- | --- | --- | --- |
| **NEC (stage ≥ Ⅱ)** | 6.0 [1.0- 35.4] | 0.049 | 6.1 [0.9- 41.3] | 0.06 |
| **Maternal hospitalization ≤ 5days** | 3.9 [0.6- 26.1] | 0.16 | 10.9 [1.0- 117.7] | 0.048 |
| **Bacteremia ≦ 10day** | 7.2 [0.7- 74.9] | 0.10 | 2.6 [0.3- 21.3] | 0.39 |

ORs and aORs with 95% CIs and p-values are presented.

**Abbreviations:** OR, odds ratio; aOR, adjusted odds ratio; CI, confidence interval; NEC, necrotizing enterocolitis.

**Table 7. Firth Logistic Regression analyses for factors associated with in-hospital mortality.**

| **Variable** | **OR [95%CI]** | **p-value** | **aOR [95%CI]** | **p-value** |
| --- | --- | --- | --- | --- |
| **NEC (stage ≥ Ⅱ)** | 4.5 [0.6- 32.0] | 0.14 | 4.7 [0.6- 35.6] | 0.13 |
| **Maternal hospitalization ≤ 5days** | 5.6 [0.99- 31.4] | 0.051 | 5.6 [0.97- 32.6] | 0.054 |
| **IVH grade Ⅲ-Ⅳ** | 5.9 [0.8- 23.8] | 0.12 | 5.6 [0.8- 34.0] | 0.12 |

ORs and aORs with 95% CIs and p-values are presented.

**Abbreviations:** OR, odds ratio; aOR, adjusted odds ratio; CI, confidence interval; NEC, necrotizing enterocolitis; IVH, intraventricular hemorrhage.

**Figure 1. Kaplan–Meier survival curves comparing neonates with and without tension pneumothorax**

Neonates who developed tension pneumothorax showed a markedly higher risk of early death, with most fatalities occurring within the first few days after birth. In contrast, those without tension pneumothorax demonstrated a more favorable survival trajectory over time. This temporal separation highlights the acute lethality of tension pneumothorax in the early neonatal period, emphasizing the need for prompt recognition and intervention.
