# Supplementary figures and images for: Mortality risk factors in actively resuscitated 22-week preterm infants: a case-control study focusing on NEC and maternal hospitalization
Source: BMC Pediatr. 2025 Oct 9;25:788. doi: 10.1186/s12887-025-06178-3 (PMC12513028; doi:10.1186/s12887-025-06178-3)

# Kaplan-Meier Survival Curve

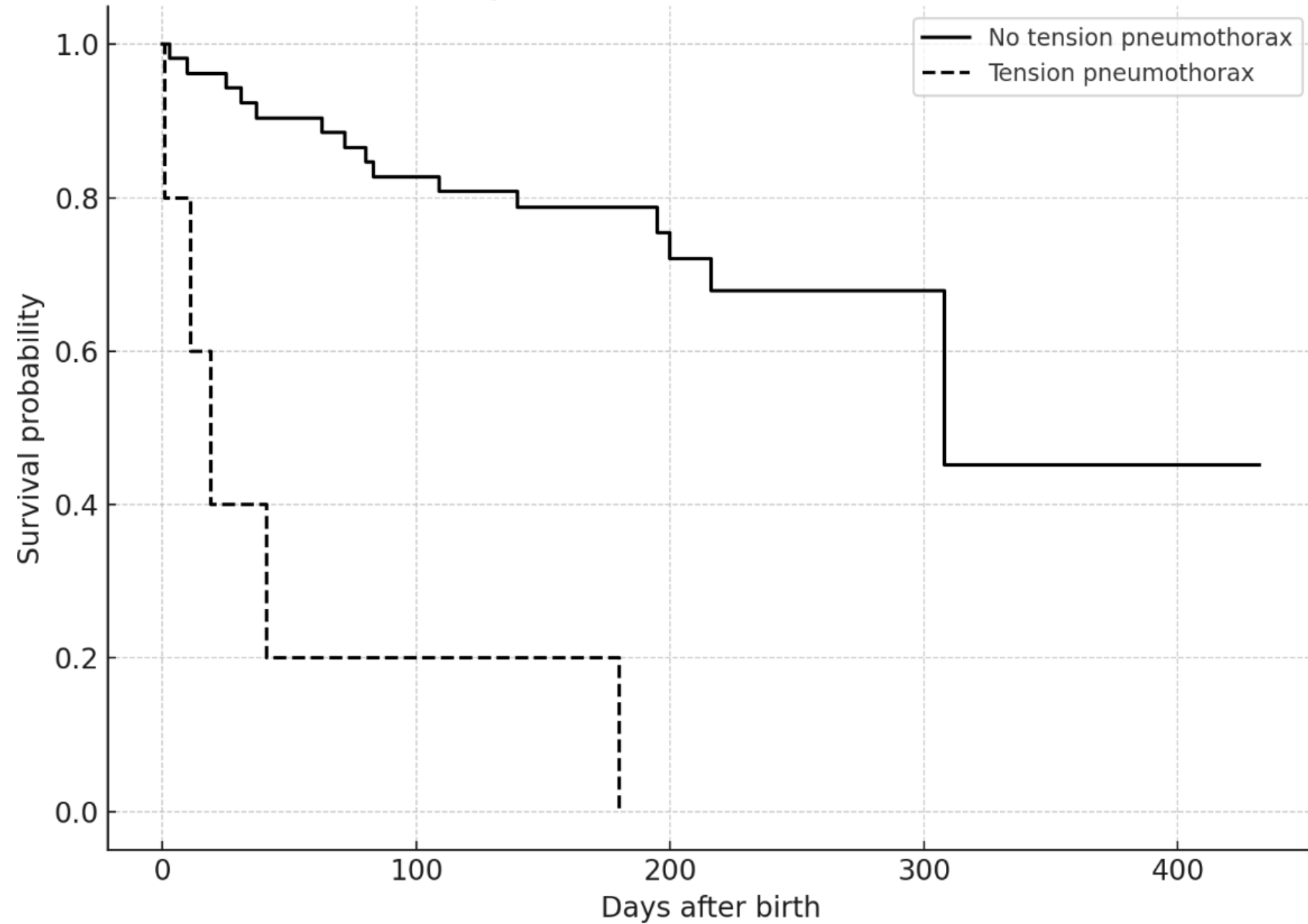

Supplement: Supplementary file 2 — Supplementary Material 2. [file 12887_2025_6178_MOESM2_ESM.pdf]
